# Hypomethylation of the Human *OAS2* Gene in Blood Cells as a Potential Biomarker for Rheumatoid Arthritis: Findings from an Iranian Case-control Study

**DOI:** 10.34172/aim.35446

**Published:** 2026-01-01

**Authors:** Delnya Gholami, Zahra Ourang, Mohammad Saatchi, Esmat Rigi Yousefabadi, Emran Esmaeilzadeh, Masoumeh Akhlaghi, Hoda Kavosi, Hamid Reza Khorram Khorshid

**Affiliations:** ^1^Genetics Research Center, University of Social Welfare and Rehabilitation Sciences, Tehran, Iran; ^2^Rheumatology Research Center, Tehran University of Medical Sciences, Tehran, Iran; ^3^Department of Internal Medicine, Arak University of Medical Sciences, Arak, Iran; ^4^Personalized Medicine and Genometabolomics Research Center, Hope Generation Foundation, Tehran, Iran; ^5^Department of Biostatistics and Epidemiology, University of Social Welfare and Rehabilitation Science, Tehran, Iran; ^6^Fetal Health Research Center, Hope Generation Foundation, Tehran, Iran

**Keywords:** Arthritis, DNA methylation, Epigenetics, Rheumatoid

## Abstract

**Background:**

Given the pivotal role of DNA methylation in the progression and severity of rheumatoid arthritis (RA), identifying a unique methylation pattern associated with the disease could support early detection, disease monitoring, and the development of personalized treatment plans. We aimed to explore the potential of *OAS2* and *OAS3* methylation levels in peripheral blood cells. These levels could be used to diagnose RA and predict patient outcomes.

**Methods:**

We collected 105 peripheral blood specimens from RA patients and 110 from healthy subjects. We then performed methylation analysis using the methylation-quantification of endonuclease-resistant DNA (MethyQESD) technique. Finally, we analyzed the data using appropriate nonparametric tests.

**Results:**

Our study revealed a significant decrease in *OAS2* gene methylation in RA patients (*P*<0.001). The ROC curve analysis further underscored the potential of *OAS2* DNA methylation (AUC=0.718, *P*<0.001) to effectively distinguish RA patients from healthy individuals. Although *OAS2* methylation was generally reduced in RA patients compared to controls, RA patients with a positive family history exhibited higher methylation than those without (*P*=0.018). Moreover, increased *OAS2* methylation was associated with reduced CRP levels (r=-0.317, *P*<0.001).

**Conclusion:**

The methylation levels of *OAS2* in peripheral blood cells show promise in distinguishing RA patients from healthy individuals. Furthermore, these levels show potential in identifying inflammation, a family history of RA, and other related disorders.

## Introduction

 Rheumatoid arthritis (RA) is a systemic and long-term disorder that causes swelling, pain, and joint stiffness.^1^ Besides the joints, the lungs and heart can also experience inflammation and related complications.^2^ Around 1% of people worldwide are affected by RA, with a female-to-male ratio of about 3:1.^3^ Twin studies show that about 50–60% of RA can be linked to genetics.^4^ RA arises from a complex interplay of genetic and epigenetic changes, environmental impacts, immune system irregularities, metabolic complications, and certain microbes, among other elements.^5^ Due to the intricate causes and processes associated with RA, high heterogeneity is observed in the disease, leading to various clinical consequences for patients. Therefore, early detection of RA and prompt treatment are crucial for an enhanced approach to managing the condition.^6^

 RA diagnosis often involves two standard tests: rheumatoid factor^7^ and anti-cyclic citrullinated peptide antibodies (anti-CCP). Anti-CCP has a sensitivity of 79% and a specificity of 93%, while RF has a sensitivity of 85% and a specificity of 69%, demonstrating their ability to forecast disease progression. Nevertheless, they could also be detected in other conditions.^8^ Thereby, researchers are diligently working to discover new and valuable indicators to aid in diagnosing and treating RA. Presently, researchers are concentrating more on uncovering indicators for identifying RA patients.

 RA is significantly associated with various epigenetic elements, such as DNA methylation patterns, histone protein modifications, chromatin remodeling, and non-coding RNAs.6 Research indicates that RA progression and exacerbation are associated with DNA methylation,^9^ and RA patients’ peripheral blood cells display unique DNA methylation patterns compared to healthy individuals. Furthermore, the specific gene methylation patterns are associated with RA’s severity and treatment options’ effectiveness.^10^

 The oligoadenylate synthetase (OAS) family genes, including *OAS2* and *OAS3*, encode proteins that play a crucial role in the body’s defense against viral infections and support various immune processes. These proteins are classified as type I interferon (IFN)-inducible genes.^11,12^ Many health conditions and disorders are related to the *OAS* gene family. They assist the body in combating viral infections,^13^ managing inflammation,^14^ and play an essential role in autoimmune disorders,^15^ cancers,^16^ and reactions to COVID-19.^17^ Their responsibilities encompass aiding in the battle against viruses, managing apoptosis and autophagy, modifying receptor activity, and transmitting signals.^18^ On the other hand, hypomethylation and up-regulation of the *OAS2* and *OAS3* genes were reported in systemic lupus erythematosus (SLE) patients,^6^ as well as Sjögren’s syndrome (SS),^19^ systemic sclerosis (SSc)^20^ and RA patients.^6^

 DNA methylation is heavily involved in managing gene expression and influencing cellular differentiation in the body. Shifts in DNA methylation patterns can offer significant clues about the beginnings and development of a disease. Additionally, identifying DNA methylation is highly dependable and precise, allowing for clear differentiation between patients and healthy individuals. ^6^

 Therefore, this investigation seeks to evaluate the methylation levels of *OAS2* and *OAS3* in blood samples to establish their efficacy as diagnostic markers for rheumatoid arthritis patients. This study was conducted in Iranian individuals using the methylation-quantification of endonuclease-resistant DNA (MethyQESD) technique. Furthermore, we examined the correlation between methylation levels on these genes and laboratory test results in patients with rheumatoid arthritis to determine if they might serve as reliable predictors of outcomes.

## Materials and Methods

###  Participants and Preparation of Samples

 This case-control study involved 105 patients diagnosed with rheumatoid arthritis, selected according to the American College of Rheumatology/European League Against Rheumatism (ACR/EULAR) classification criteria^21^ and the updated European League Against Rheumatism (EULAR) management recommendations.^22^ Moreover, 110 healthy volunteers were recruited as the control group. Patients with RA and healthy individuals were enrolled from the rheumatology clinics at Shariati Hospital in Tehran, Iran, from 2019 until 2022. Healthy participants were recruited from individuals attending the hospital for routine check-ups. All control subjects were examined by a rheumatologist and confirmed to have no symptoms and no personal or family history of RA or other autoimmune disorders. Individuals with any other rheumatic or autoimmune diseases were excluded.

 We collected data on the participants, including their gender, age, systolic and diastolic blood pressure (SBP), height, and weight, to calculate their body mass index (BMI). We also examined whether anyone in their family had RA or other autoimmune diseases. Various lab tests, such as C-reactive protein (CRP), erythrocyte sedimentation rate (ESR), white blood cell (WBC) count, hemoglobin, creatinine, platelet count (PLT), blood urea nitrogen (BUN), fasting blood sugar (FBS), triglycerides (TG), high-density lipoprotein (HDL), and low-density lipoprotein (LDL) were recorded. The participants’ personal details and lab findings in this study were obtained through a standardized questionnaire. The clinical stage of the disease, disease activity, the number of painful and swollen joints and treatment regimens were generally similar among patients. Although individual-level data were not available, a trusted doctor assured us that the clinical stage of the disease and treatment regimens were the same for all patients.

 Finally, approximately 5 mL (milliliters) of peripheral whole blood was drawn from every person and transferred into specific EDTA anticoagulant tubes (Kima Teb, Tehran, Iran). The tubes were then kept at -20°C for later use.

###  DNA Extraction

 We employed the PrimePrep Genomic DNA Isolation Kit (ARTOUN, Iran) to isolate genomic DNA from whole blood cells. We evaluated the quantity, quality, and integrity of the isolated DNA with a NanoDrop 2000c spectrophotometer (Thermo Fisher Scientific, USA) and agarose gel electrophoresis (Merck, Germany).

###  Methylation Analysis by MethyQESD

 Promoter methylation levels of *OAS2* and *OAS3* were quantified using the Methylation-Quantification of Endonuclease-Resistant DNA (MethyQESD) method, as previously described in detail by Rigi Yousefabadi *et al*., 2025. In brief, MethyQESD relies on differential digestion of genomic DNA by methyl-sensitive and methyl-insensitive restriction enzymes followed by quantitative real-time PCR. ^23^

###  DNA Digestion

 Genomic DNA was independently digested using the methyl-sensitive enzyme *Hin6I* (Thermo Fisher Scientific, Waltham, MA, USA), which selectively cleaves unmethylated GCGC sites within the promoter regions. Parallel calibration digestions were performed using the methyl-insensitive enzymes *XbaI* (Thermo Fisher Scientific, Waltham, MA, USA) and *DraI* (Thermo Fisher Scientific, Waltham, MA, USA), which generate reference fragments without affecting the methylation status. Digestions were carried out according to the manufacturers’ instructions.

###  Real-Time PCR

 The quantity of methylated DNA resistant to breakdown by the enzyme *Hin6I* was assessed through real-time PCR (StepOne, Applied Biosystems, Foster City, CA, USA). This measurement is calibrated compared to a reference DNA that is not cleaved, derived from the CalD reaction. Table 1 lists the primers utilized for amplifying the DNA segments near the CpG site in the promoters of the *OAS2* and *OAS3* genes.

**Table 1:** Selected promoter regions and primers designed for the *OAS2* and *OAS3 *genes

**Gene**	**Sequences**	**Primers**	**Product size**
*OSA2*	CCTGCTTCTAAGCACTTTGCAGGTATTATTTAATCATCAAGCTAATCCTACGAGA GAGCTGCCATTTTCCCCCAGCTTACAGATGGGGGAATTGAGGCGCGAAGAGGG CAGGCGATGTGCTCAAGGACAGACATCTAGCAGGTATGAAGCCCTCACAATGGGGT	F: 5’-AATCCTACGAGAGAGCTGCC-3’ R: 5’-ACCTGCTAGATGTCTGTCCT-3’	100 bp
*OSA3*	GGAATTTCCCAAGTTTGGGGAAGACAGGAACTGCAGCGCCCCTCCCCGTTTCACGC CACGCGCGGGACCGAGGACCTAGGACCTGGCCAGCTGGGCGTGGTTCGGAGAG CCGGGCGGGAAAACGAAACCAGAAATCCGAAGGCCGCGCCAGAGCCCTGCTTC CCCTTGCACCTGCGCCGGGCGGCCATGGACTTGTACAGCACCCCGGCCGCTGC	F: 5’-TTGGGGAAGACAGGAACTGC-3’ R: 5’-GGGGTGCTGTACAAGTCCAT-3’	192 bp

 Every real-time PCR test utilized a combination of 1.5 µL of digested DNA, 0.5 µL each of forward and reverse primers (see Table 1), along with 5 µL of 2 × SYBR Green PCR Master Mix (Thermo Fisher Scientific, Waltham, MA, USA), summing up to 10 µL in total. Each reaction was performed in duplicate. The process for real-time PCR for the two genes began with 5 minutes at 95˚C to denature the DNA. Then, it went through 40 cycles: first, 20 seconds at 95˚C, then 20 seconds at 63.5˚C, and finally, 20 seconds at 72˚C.

 To assess the melting curve, the temperature was increased from 50 °C to 95 °C in gradual increments. This step was performed to verify that the size of the PCR products is accurate and that there are no non-specific PCR products. Also, we evaluated real-time PCR products with an agarose gel electrophoresis 2% (Merck, Germany) (Supplementary Figure S1). To assess the extent of promoter methylation, we analyzed the differences in Ct-values between MQD and CalD according to the formula below.

 Methylation% = 2^Ct Calibrator−Ctmethylationquantification^ × 100

 A detailed description of the MethyQESD workflow, primer design strategy, and enzymatic recognition sites is provided in our previous publication.^23^

###  Statistical Analysis

 Statistical analysis was done using GraphPad/Prism5/PrismDemo (GraphPad Software, USA) and SPSS23 (IBM Corp., Armonk, NY, USA). To evaluate and contrast groups based on essential characteristics, and clinical and lab results, we utilized a range of statistical tests, including the chi-square test, Student’s* t*-test, and Mann-Whitney U-test, to obtain P values. Before performing the chi-square test, its underlying assumptions were examined; no expected cell count was less than 1, and fewer than 20% of the expected cell counts were below 5. All real-time tests were duplicated, and the outcomes were reported as the mean ± standard deviation (SD). Since the number of the studied samples was more than 50, the Kolmogorov-Smirnov test and graphical methods, including Q–Q plots were utilized to analyze how the results were distributed (parametric or non-parametric). Due to the non-normal distribution of *OAS2* and *OAS3* within both patients and healthy groups, we employed the Mann-Whitney U-test to assess these changes between the patients and healthy individuals.

 To assess effectiveness, we analyzed the ROC curve and determined the areas beneath it (AUC). This test identified the optimal cutoff value of DNA methylation for rheumatoid arthritis diagnosis, and subsequently, sensitivity and specificity values were determined. Additionally, Spearman’s rank correlation test was performed to evaluate the relationship between the percentage of methylation in the *OAS2* and *OAS3* promoters and various laboratory characteristics (e.g. CRP, ESR). In statistical analysis, a *P*-value less than 0.05 was considered significant.

## Results

###  Patient Characteristics 

 The demographic, baseline, and clinical characteristics of patients with rheumatoid arthritis and healthy controls are summarized in Table 2. No significant differences were found between the two groups in terms of age or sex distribution. Laboratory and clinical parameters are also presented in Table 2.

**Table 2:** Baseline and Laboratory characteristics of patients (RA) and control subjects who participated in the study

**Characteristics or Lab Tests**	**Controls (** ***n*** **=110)**	**RA (** ***n*** **=105)**	***P*** ** Value**
Age (mean ± SD)	44.06 ± 11.49	46.84 ± 10.41	0.064
Gender *n* (%)			0.647
Male	28 (25.5%)	30 (28.6%)	
Female	82 (74.5%)	75 (71.4%)	
Age of onset (mean ± SD)	—	41.32 ± 10.44	—
BMI (mean ± SD)	24.06 ± 3.44	26.21 ± 2.43	< 0.001
SBP (mean ± SD)	120.59 ± 9.85	122.33 ± 12.84	0.267
DBP (mean ± SD)	79.18 ± 8.44	78.28 ± 7.96	0.425
Positive family history n (%)	0	17(16.2%)	—
ESR (mm/hr)	15.70 ± 7.02	30 (21.5-47)	< 0.001
CRP (mg/l)	3 (2.5-4.4)	14 (8.7-21.1)	< 0.001
White blood cell (10^9^/L)	6489.90 ± 1466.78	7274.76 ± 2227.71	0.003
Hemoglobin	14.22 ± 1.47	12.39 ± 1.06	< 0.001
PLT(10^9^/L)	241.44 ± 66.58	261.93 ± 73.73	0.033
Creatinine (mg/dL)	0.87 ± 0.19	1.04 ± 0.17	< 0.001
BUN	15.91 ± 4.68	17.31 ± 4.69	0.030
FBS	90.88 ± 18.94	96.33 ± 16.31	0.025
HDL	49.81 ± 11.96	48.82 ± 7.39	0.465
LDL	105.54 ± 37.61	111.70 ± 29.50	0.184
TG	152.17 ± 58.18	167.58 ± 48.51	0.037

*P* value < 0.05. Data are presented as mean ± SD for normally distributed variables, median (IQR) for skewed variables, or n (%). RA, Rheumatoid arthritis; BMI, Body mass index; SD, Standard deviation; SBP, Systolic blood pressure; DBP, Diastolic blood pressure; ESR, Erythrocyte sedimentation rate; CRP, C-reactive protein; BUN, Blood urea nitrogen; PLT, Platelet; HDL, High density lipoprotein; LDL, Low density lipoprotein; TG, Triglyceride; FBS, Fasting blood sugar.

###  OAS2 and OAS3 Methylation

 Our analysis examined the methylation levels of the *OAS2* and *OAS3* genes in patients with rheumatoid arthritis (RA) compared with healthy individuals. The average promoter methylation level of *OAS2* was significantly lower in RA patients (23.09 ± 23.96) than in controls (39.50 ± 24.45), with a mean difference that remained statistically significant (95% CI: 11.9–24.2; *P* < 0.0001). No meaningful difference in *OAS2* methylation was observed between RA patients with an age of onset ≤ 35 years and those with onset > 35 years (Table 3).

**Table 3:** Percentages of methylation in the *OAS2* and *OAS3* genes in cases and control groups and their diagnostic value

**Genes**	**AM % in** **Control (** ***n*** **: 110)**	**AM % in** **RA (** ***n*** **: 105)**	***P***	**Cutoff**	**Sensitivity**	**Specificity**	**AM% in age of onset ≤ 35**	**AM% in Age of onset > 35**	***P*** **Value**
*OAS2*	39.50 ± 24.45	23.09 ± 23.96	< 0.0001 (95% CI: 11.9–24.2)	25.88	75.23%	70.00%	24.43 ± 23.56	22.44 ± 24.29	0.692
*OAS3*	25.21 ± 20.58	22.12 ± 20.55	0.273 (95% CI: −1.37 to 6.56)	—	—	—	21.88 ± 17.91	22.24 ± 21.82	0.935

AM: Average Methylation.

**Figure 1:**
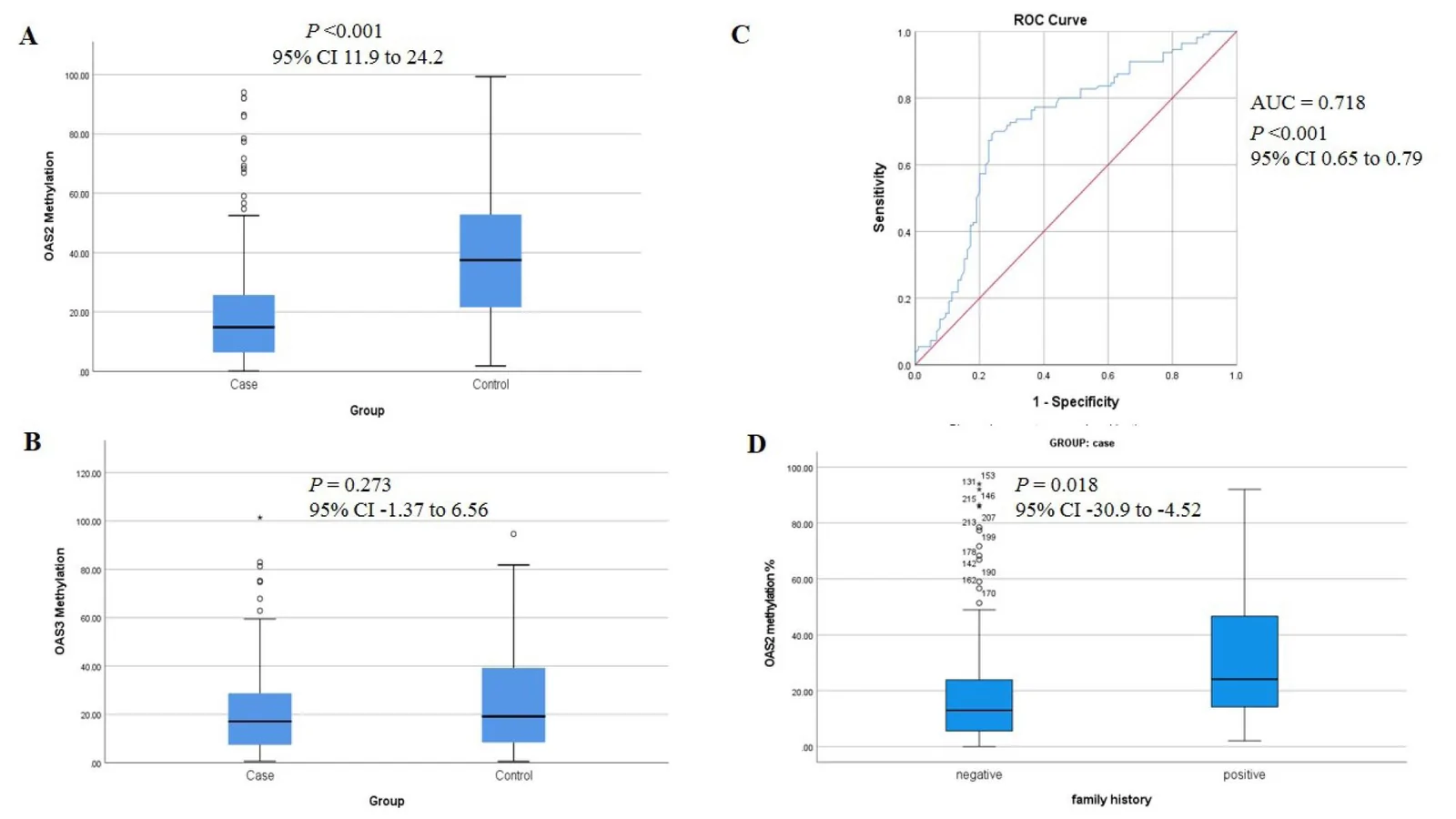
Promoter methylation levels of *OAS2* and *OAS3* in rheumatoid arthritis (RA) patients and healthy controls. **(a)** Comparison of *OAS2* promoter methylation between RA patients and controls. **(b)** Comparison of *OAS3* promoter methylation between RA patients and controls. **(c)** Receiver operating characteristic (ROC) curve for *OAS2* promoter methylation in discriminating RA patients from controls (AUC = 0.718; 95% CI: 0.65–0.79; *P* < 0.001). **(d)** Comparison of *OAS2* promoter methylation between RA patients with and without a family history of RA or other autoimmune diseases. Outliers in box-and-whisker plots (Figures 1a, 1b, and 1d) were defined as values exceeding 1.5 × IQR above the upper quartile or below the lower quartile. Extreme outliers were defined as values exceeding 3 × IQR.

 The RA group also showed a lower mean methylation level of *OAS3* compared with healthy participants; however, this difference was not statistically significant (95% CI: −1.37 to 6.56; *P* = 0.273) (Table 3). Figures 1a and 1c depict the distribution and group comparisons of *OAS2* and *OAS3* promoter methylation levels.

 Additionally, the diagnostic value of the *OAS2* promoter methylation level for RA was shown through ROC analyses (AUC = 0.718; 95% CI: 0.65–0.79; *P* < 0.001). According to our results, the most effective cutoff point for *OAS2* promoter methylation level for differentiating RA patients from healthy individuals was ≤ 25.88% with a sensitivity of 75.23% and a specificity of 70.00% (Figure 1b and Table 3).

###  Association of OAS2 Methylation with Clinical Traits

 We further assessed the association between *OAS2* promoter methylation and key clinical characteristics of RA. Logistic regression–based comparisons showed that RA patients with a positive family history of RA or other autoimmune diseases had higher *OAS2* methylation levels (32.13 ± 24.18) than those without such a background (21.34 ± 23.66). This difference was statistically significant (95% CI: −30.9 to −4.52; *P* = 0.018), although both subgroups demonstrated lower methylation levels compared with healthy controls (Figure 1d and Table 4).

**Table 4:** Association of *OAS2* promoter methylation level with various parameters in RA patients

**Methylation**	**Mean of ** ***OAS2*** ** Promoter Methylation** **Median (IQR)**	***P*** ** Value**
Family history		
Negative	24.65 (9.94‒48.63)	0.018
Positive	24.49 (14.4‒49.57)	
CRP (mg/L)		
Negative ( < 6)	37.37 (21.28‒54.15)	< 0.001
Positive ( ≥ 6)	13.26 (5.67‒24.7)	
ESR(mm/hr)		
Negative ( ≤ 20)	37.31 (17.2‒53.22)	
Positive ( > 20)	17.19 (7.36‒34.04)	0.09

Data are presented as mean ± SD for normally distributed variables, median (IQR) for skewed variables, or n (%). *P* value < 0.05. SD, Standard deviation; RA, Rheumatoid arthritis; CRP, C-reactive protein; ESR, Erythrocyte sedimentation rate.

 Regarding inflammatory markers, RA patients with elevated CRP levels ( ≥ 6 mg/L) exhibited markedly lower *OAS2* methylation (17.56 ± 19.38) compared with those with CRP < 6 mg/L (45.19 ± 28.05). This difference was statistically significant (95% CI: −45.4 to −16.2; *P* < 0.001). Similarly, patients with ESR ≥ 20 mm/hr had lower methylation levels (20.92 ± 23.88) than those with ESR < 20 mm/hr (30.38 ± 23.25). However, this difference did not reach statistical significance (95% CI: −22.1 to −3.7; *P* = 0.09) (Table 4).

###  Correlation Analysis

**Figure 2:**
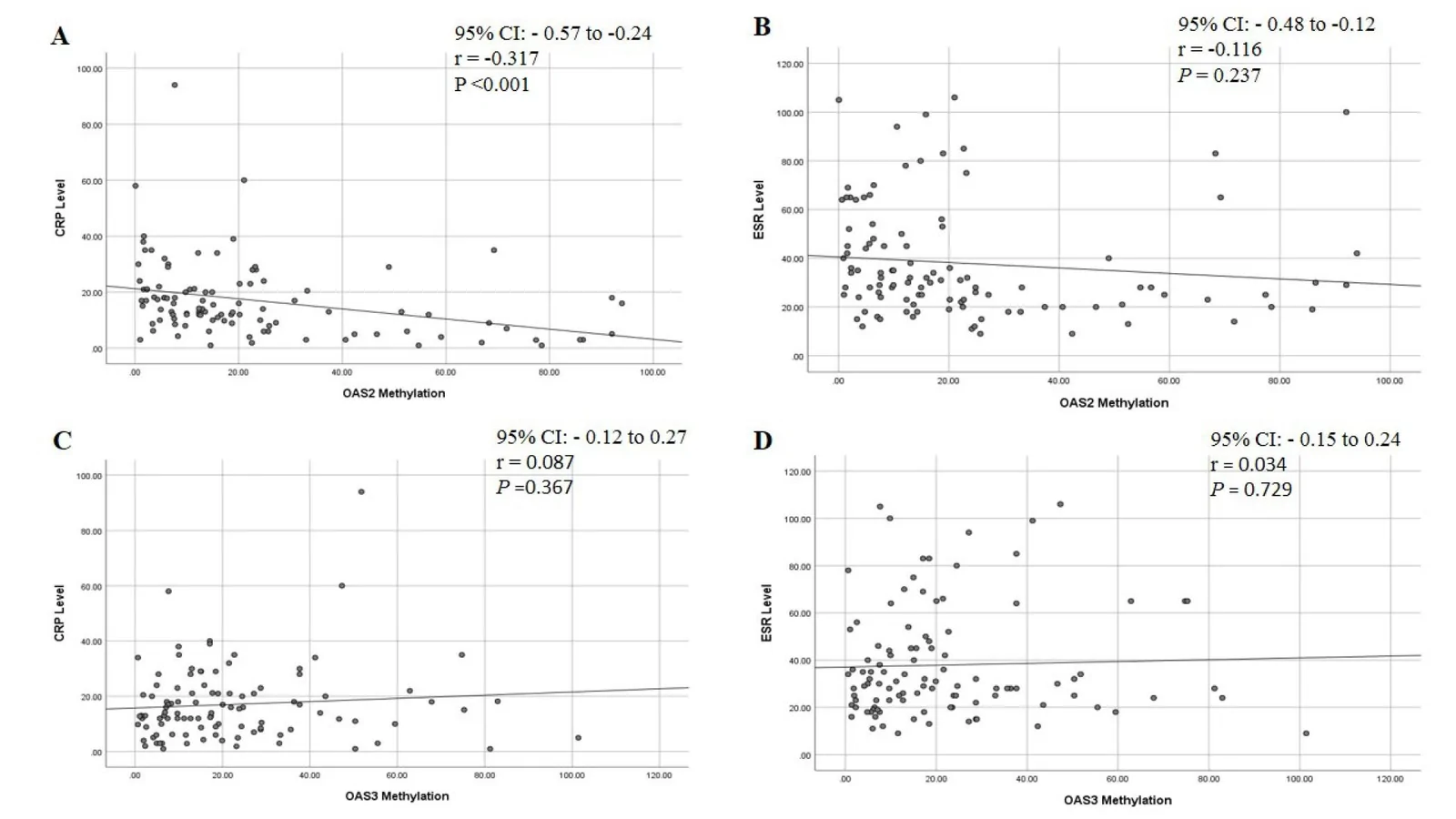
Correlation analyses in patients with rheumatoid arthritis (RA). **(a)** Correlation between *OAS2* methylation and C-reactive protein (CRP) levels. **(b) **Correlation between *OAS2* methylation and erythrocyte sedimentation rate (ESR). **(c)** Correlation between *OAS3* methylation and CRP levels. **(d)** Correlation between *OAS3* methylation and ESR

 Correlation analysis demonstrated a significant inverse association between *OAS2* promoter methylation and serum CRP levels in RA patients (r = −0.317; 95% CI: −0.57 to −0.24; *P* < 0.001). In contrast, no significant correlation was observed between *OAS2* methylation and ESR values (r = −0.166; 95% CI: −0.48 to −0.12; *P*= 0.237) (Figure 2a and 2b). Similarly, methylation of the *OAS3* promoter showed no significant relationship with either CRP or ESR levels in RA patients (Figure 2c and 2d).

## Discussion

 Our study analyzed the methylation status of the promoters for the *OAS2* and *OAS3* genes, employing the MethyQESD method. Previous studies have confirmed that the MethyQESD method is robust, rapid, reliable, and easy to use for evaluating methylation levels. It also does not need a significant amount of DNA.^24-27^ Most prior studies relied on bisulfite treatment to assess patterns of methylation. However, this process is costly and time-intensive and can potentially damage the extracted DNA.^28^

 RA is an autoimmune, multifactorial, and systemic disorder.^1^ Epigenetic factors, particularly DNA methylation, contribute to understanding the causes of RA.^29^ There are noticeable variations in RA patients’ methylome compared to unaffected individuals.^30^ Early recognition and management of RA patients could positively impact their health and quality of life. DNA methylation–based diagnosis and prediction have been applied in various diseases and have produced promising preliminary results.^31^

 For the first time in Iranian individuals, this study investigated the methylation status of promoter regions of the *OAS2* and *OAS3* genes in both RA and healthy individuals. Our results revealed a significant difference in methylation levels of *OAS2* between RA and control groups but not in methylation levels of *OAS3*.

*OAS2* participates in the IFN-β signaling process and is considered a potential gene related to RA.^32^ Research indicated that *RSAD2, OAS2*, and *MX1* could reliably predict the treatment response of individuals with RA.^33^ On the other hand, higher levels of *OAS2* and *OAS3* have been associated with lupus nephritis, dermatological manifestations of discoid lupus, and active SLE in patients with renal involvement and arthritis.^6,34-36^ Furthermore, *OAS1, OAS2*, and *OASL* have been proposed as valuable markers for differentiating SLE patients from those who are healthy.^6^ Other research has indicated that *OAS3* levels in B cells can help diagnose male SLE patients. ^37^ Likewise, the *OAS2* gene showed hypomethylation and overexpression in SLE patients.^38^ Another study revealed a strong correlation between the level of methylation in the *OAS2* gene and the activity of RA disease in CD4 + T cells.^39^ Similarly, in limited samples taken from the salivary glands and blood cells of individuals with Sjögren’s syndrome, *OAS2 *showed reduced methylation and increased expression.^19^ Furthermore, other investigations have revealed that *OAS3* shows reduced methylation in CD14 + CD16- monocytes and CD4 + T cells from patients with SSc who were African American or of Spanish descent.^20,40^

 The ROC curve analysis indicated that *OAS2* (AUC = 0.718) methylation levels can effectively differentiate RA patients from healthy controls.

 Consistent with our research, the methylation status of other genes has been investigated in several different samples as a biomarker in the timely diagnosis of rheumatoid arthritis. For example, Fang *et al*. proposed *OAS1*, *OAS2*, and *OASL* as high-sensitivity diagnostic biomarkers with AUC > 0.70 to differentiate SLE from healthy persons. Contrary to our findings, they observed no difference in these genes’ expression and methylation levels in RA subjects.^6^ According to M. Salesi *et al*., the *IFI44L *promoter’sDNA methylation level was a useful potential diagnostic biomarker for differentiating between SLE (AUC = 0.817) and RA (AUC = 0.842) patients and healthy controls.^24^ In line with this study, Zhao and colleagues revealed that the *IFI44L* promoter methylation could differentiate RA from primary Sjögren’s syndrome and SLE from healthy individuals.^41^ According to results from another study, the methylation level of *HTR2A* cg15692052, when combined with various clinical characteristic variables, could differentiate RA patients with AUCs from 0.672 to 0.757 and seronegative RA patients with AUCs from 0.825 to 0.966 for RF/CCP double-negative and AUCs from 0.928 to 0.932 for RF-negative patients.^42^ *HIPK3* methylation has also been reported as a potential diagnostic biomarker for RA with an AUC of 0.829. However, an enhanced AUC of 0.864 was obtained in a prediction model that included *HIPK3*, ACPA, and RF.^43^ Gang Fang *et al*. reported *BCL11B*, *CCDC88C*,* FCRLA*, and *APOL6* as putative biomarkers of RA using a comprehensive study of gene expression and DNA methylation datasets. The development of RA is significantly influenced by these genes, which are linked to differentially methylation sites.^44^ More recently, Muciente *et al.* demonstrated that differential methylation of specific CpG sites could reliably aid RA diagnosis and assess disease severity.^45^ Additionally, a recent EPIC-array based EWAS on treatment-naïve, ACPA-positive RA patients identified thousands of differentially methylated CpG sites and DMRs, highlighting novel candidate loci.^46^ Another study proposed a methylation-based classification algorithm showing promising diagnostic accuracy for RA.^47^ Together, these findings, along with our results on *OAS2*/*OAS3* promoters, reinforce the potential of DNA methylation as a biomarker for RA. They also emphasize the need for validation in larger cohorts and longitudinal studies.

 In this study, the methylation level of *OAS2* was negatively correlated with CRP. This finding indicated a considerable correlation between *OAS2* methylation and CRP levels (r = -0.317, *P* = 0.001). The levels of CRP and ESR reflect systemic inflammation and tissue damage. These are common indicators of inflammation that contribute to acute and chronic inflammatory conditions and RA.^48^ Inflammation significantly contributes to the development and progression of RA.^49^ The activation, proliferation, growth, and development of T and B cells by interleukins (IL) are critical in the body’s response to inflammation. For example, *IL-1* and *IL-6* contribute to regulating bone deterioration in cases of RA.^51^ Also, RA individuals have elevated levels of *IL-23*, which correlates with increased CRP levels.^51^ *OAS2*, *OAS3*, and *OASL* contribute to inflammation induced by IFN-α2a, even in the absence of viral or bacterial infection, and have been implicated in active cases of SLE.^34^ Therefore, the role of inflammation in the pathogenesis of RA is undeniable. Thus, it can be stated that *OAS2* methylation levels in individuals with RA are connected to CRP and can provide insight into the progression of inflammation. These results provide insight into how inflammation affects *OAS2* expression and methylation in RA.

 Having a family history increases the risk of developing RA significantly, raising the chances by two to four times for close relatives and having significant genetic overlap with similar autoimmune conditions. The heritability of RA is estimated at approximately 40%, and this proportion is not influenced by gender. Still, individuals with seropositive RA have a higher chance of its heritability compared to seronegative RA. Information about family history may assist in diagnosing RA, predicting disease progression, and assessing treatment effectiveness; however, the evidence remains inconclusive.^52^ This study found that RA patients with relatives affected by RA or other autoimmune diseases exhibited significantly higher *OAS2* promoter methylation compared with those without a family history of RA or autoimmune conditions (*P* = 0.018).

 Different stimuli regularly alter DNA methylation patterns and can be transmitted to daughter cells during cell replication.^53^ Therefore, we cannot provide a clear explanation for the association between *OAS2* gene methylation patterns and family history. This is because differences in *OAS2* methylation patterns cannot be attributed solely to environmental factors or to the individual’s genetic background. Thus, elucidating this relationship is challenging and requires more comprehensive research.

 Another critical question in epigenetic research is whether the changes in the epigenome linked to a disease happen before the disease emerges or if they are induced by the disease’s progression.^54^ This raises the possibility that differences in *OAS2* gene methylation patterns may represent epigenetic changes that occur before disease onset. However, recognizing abnormal patterns of DNA methylation before disease onset could provide new insight into risk factors and their underlying causes, and may help identify biomarkers for diagnosis, disease course, and treatment response.^54^

 The findings of our research suggest that methylation levels in the *OAS2* gene could be a crucial marker for identifying RA cases. Still, this research contains some shortcomings that need to be solved in the future. More exploration is necessary to understand the effects of *OAS2* DNA methylation on gene expression. Unfortunately, gene expression data were not available for these samples. This complicates our ability to assess the impact of reduced DNA methylation on gene expression and its implications for RA. Each type of cell exhibits unique methylation patterns. Therefore, findings from heterogeneous cell groups may influence the results and comprehension of studies focused on whole blood methylation.^54^

 Our results revealed only a slight correlation, indicating that additional data with larger sample sizes are needed for validation. Therefore, conducting similar studies with more diverse populations is recommended for more reliable outcomes. It remains uncertain whether this alteration in DNA methylation of the *OAS2* promoter occurred before the onset of the disease or if it is a consequence of the disease’s progression. Therefore, monitoring the progression of diseases through long-term studies would provide more valuable insights. In addition, detailed quantitative data on disease activity (mild, moderate, severe) were not available for all patients due to limitations in clinical records. However, all patients were evaluated by a rheumatologist and were in comparable stages of disease; none were in the early clinical stage.

## Conclusion

 Our analysis revealed that the average methylation of the *OAS2* promoter in individuals with rheumatoid arthritis was significantly lower than in healthy controls, and ROC analysis indicated a potential diagnostic value (AUC = 0.718, *P* < 0.001). Furthermore, *OAS2* promoter methylation was inversely correlated with CRP levels in RA patients and was higher in those with a positive family history compared to those without. These findings suggest that *OAS2* promoter methylation may provide useful information regarding disease activity and family history in RA. However, since this study only included RA patients and healthy controls, further research is needed to determine whether these methylation changes are specific to RA or also occur in other autoimmune diseases.

## Supplementary File

 Representative banding patterns of the OAS2 and OAS3 genes before and after the MQD and CalD reactions. The banding patterns obtained before enzymatic digestion and after the CalD reaction are similar, whereas the MQD reaction generates distinct fragment patterns due to enzyme-specific restriction cleavage sites, resulting in digestion into fragments of different sizes.
