# Supplementary figures and images for: Hypomethylation of the Human OAS2 Gene in Blood Cells as a Potential Biomarker for Rheumatoid Arthritis: Findings from an Iranian Case-control Study
Source: Arch Iran Med. 2026 Jan 1;29(1):12–20. doi: 10.34172/aim.35446 (PMC13338500; doi:10.34172/aim.35446)

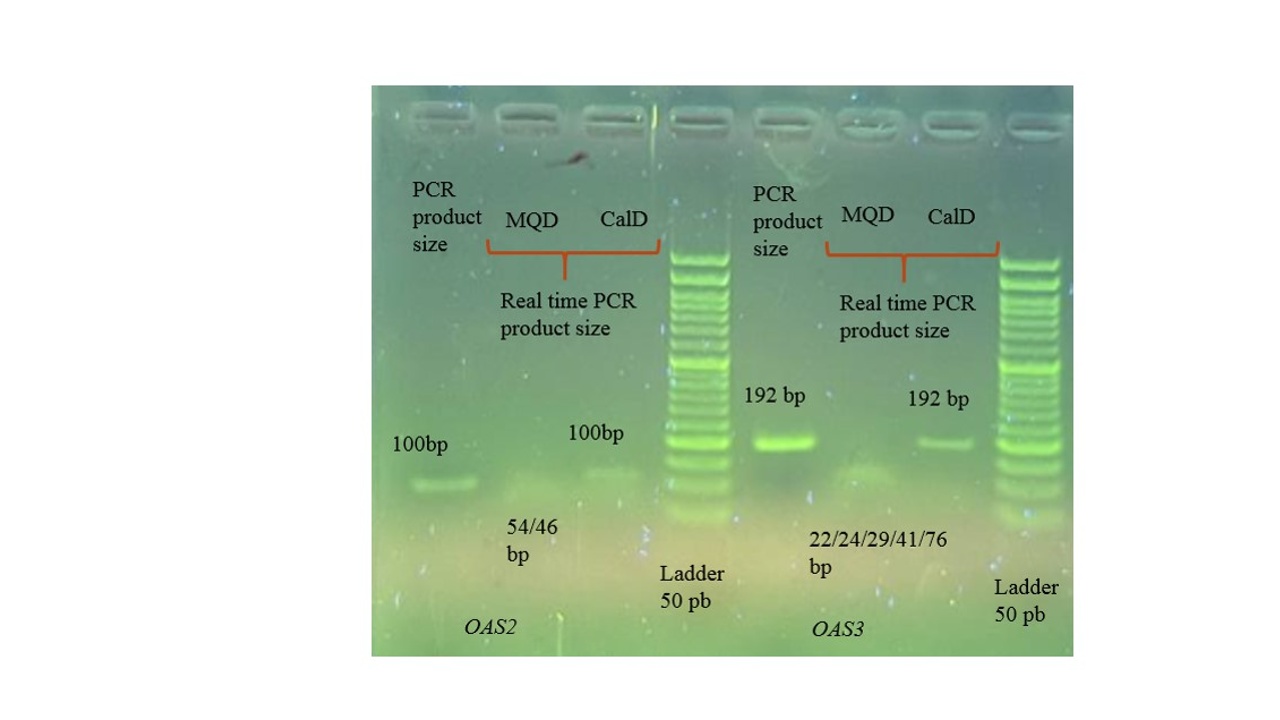

Supplement: Supplementary file 1 — contains Figure S1. [file aim-29-12-s001.jpg]
