# Supplementary material for: Hypomethylation of the Human OAS2 Gene in Blood Cells as a Potential Biomarker for Rheumatoid Arthritis: Findings from an Iranian Case-control Study
Source: Arch Iran Med. 2026 Jan 1;29(1):12–20. doi: 10.34172/aim.35446 (PMC13338500; doi:10.34172/aim.35446)
Supplement: Supplementary file 2 [file aim-29-12-s001.pdf]

Arch Iran Med. January 2026;29(1):12-S1

doi:10.34172/aim.35446

http://journalaim.com

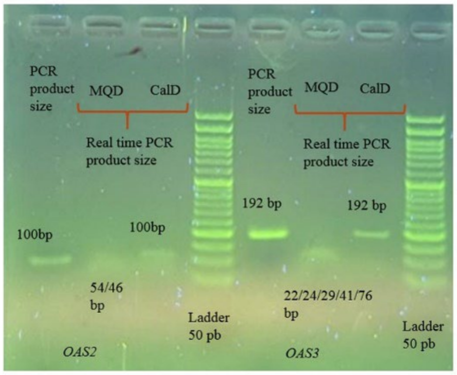

Figure S1. Representative banding patterns of the OAS2 and OAS3 genes before and after the MQD and CalD reactions. The banding patterns obtained before enzymatic digestion and after the CalD reaction are similar, whereas the MQD reaction generates distinct fragment patterns due to enzyme-specific restriction cleavage sites, resulting in digestion into fragments of different sizes.
